# The Effect of Weaning and Slaughter Age on the Physicochemical and Sensory Characteristics of *Arouquesa* Beef—A PDO Portuguese Meat

**DOI:** 10.3390/foods11162505

**Published:** 2022-08-19

**Authors:** José António Silva, Ricardo Cardoso, Raquel Vieira, José Carlos Almeida, Maria José Gomes, Carlos Venâncio, Luis Patarata

**Affiliations:** 1 AL4Animals—Associate Laboratory for Animal and Veterinary Sciences, CECAV–Veterinary and Animal Research Centre, Universidade de-Trás-os-Montes e Alto Douro, 5000-801 Vila Real, Portugal; 2 CITAB, Centre for the Research and Technology of Agro-Environment and Biological Sciences, Universidade de-Trás-os-Montes e Alto Douro, 5000-801 Vila Real, Portugal

**Keywords:** consumer, CATA, consumer information autochthonous breeds meat, weaning age, slaughter age

## Abstract

(1) Background: Autochthonous breeds meat is well accepted due to its sensory characteristics, perceived low environmental impact, and animal welfare. We aimed to evaluate the effect of weaning and slaughter age on the physicochemical and sensory characteristics of *Arouquesa*, a Portuguese Protected Designation of Origin (PDO) meat and to evaluate the psychological effect of knowing the weaning age on the consumer’s hedonic evaluation. (2) Methods: Meat from 26 animals was assigned to 4 groups, with combinations of weaning (W) at 9 or 5 months and slaughter (S) at 9 or 12 months: W9-S9, W9-S12, W5-S9, and W5-S12. The meat was analysed for pH_24h_, colour (L*a*b*), cooking losses and shear force. A Check All that Apply test was made with 70 consumers; they were also asked to punctuate the hedonic appreciation of anonymous and weaning age-identified meat. (3) Results: W9-S9 were more tender, had lower shear force, and was juicier than W5-S9. When animals were slaughtered at 12 months, there were no differences in the physicochemical and sensory characteristics between the weaning ages. The effect of information about the weaning age influences the consumer’s hedonic evaluation, as revealed by the comparison between the anonymous and identified samples. (4) Later weaning resulted in more tender meat when the slaughter was at 9 months and positively impacted consumer perception.

## 1. Introduction

The beef sector is under considerable pressure. Red meats are assumed by some empirical studies and by consumers’ as nutritionally unhealthy and mainly associated with non-communicable diseases triggered by an unbalanced diet [1]. That perception was aggravated by the International Agency for Research on Cancer report that considered red meat a probable cause of colon cancer [2,3]. Additionally, a particular segment of consumers, particularly those from urban contexts with purchasing power, are concerned with the environmental impact of bovine production [4,5]. Consumers with a higher literacy understand that beef obtained from extensive production and the animals’ consumption of local feed has a lower environmental impact [6]. These same consumers from the wealthier regions, with purchasing power to buy beef regularly, are also concerned with animal welfare. These two consumer concerns grow hand-in-hand once both constructs are deeply interrelated [7,8]. The negative view of beef production is highly associated with intensive production, high energy inputs in the circuit of the cereal-soya-based feed, and animal welfare and with closure, high densities, and early separation of the calves from the mothers, among other factors [9]. Extensive production based on grazing fresh pasture, exercise in large spaces, and maintaining the calves with their dams with natural milk feeding is positively seen by the consumer, independently of its effective impact on the environment and the animal’s welfare [10,11]. Autochthonous beef breeds, usually raised in traditional conditions, with grazing in mountain regions, meet most of the concerns of the modern consumer, both on tangible meat quality traits, such as marbling, tenderness, and flavour, and in psychological perceptions of extrinsic factors, such as welfare and environmental impact [11,12]. Furthermore, raising autochthonous breeds has a substantial socio-economic impact on rural communities, represents a part of these regions’ culture and traditions and contributes to the preservation of genetic patrimony [13]. When raised in extensive pasturage in mountain areas, they contribute to the regulation of spontaneous vegetation, avoiding the overgrowth of certain species that contribute to the wildland fire risk [14]. Despite the potential advantages of autochthonous breeds, the low productivity limits the interest in its production and has contributed to reducing the number of raised animals [15].

To avoid fraud and to increase consumer confidence, it is common to have Protected Designation of Origin (PDO) certification schemes associated with autochthonous beef meat [16]. *Arouquesa* is an autochthonous Portuguese breed with rustic animals produced under traditional agriculture systems. In the past, these animals were used for their workforce and meat production. The production region has moderate summer temperatures and abundant water until the end of spring, which allows grazing throughout almost the entire year [17]. The meat is obtained from animals slaughtered between 9 months and 1 year. Once there is no commercial interest in the milk, animals are suckled for an extended period, supplemented with pasturage, hay, and cereal-based feed.

Meat quality is a complex concept and can be defined as the characteristics of meat that satisfy consumers. The quality concept can be divided into intrinsic quality traits, such as shape, colour, tenderness, juiciness, flavour, and nutritional properties, and extrinsic quality traits, such as price, brand, or quality label [5].

The slaughter and the weaning age are among the several antemortem and postmortem factors potentially affecting the quality of veal meat [18,19]. Late weaning may benefit not only the sensorial quality of meat but also beneficial health characteristics related to a higher PUFA/SFA ratio [20] and a lower n-6/n-3 ratio [21,22]. Since calves are born pseudo-monogastric, without a functioning rumen, late weaning is also advantageous for calves since it allows a progressive transition from milk to solid feed, allowing higher solid intakes before, during, and after weaning [23]. Veal is widely prized by consumers mainly for its healthiness perception, particularly in animals reared on pasture with natural suckling [22].

This work aimed to evaluate the effect of weaning and slaughter age on the physicochemical and sensory characteristics of *Arouquesa* meat assessed by a Check All That Apply (CATA) test made with consumers and to evaluate the psychological effect of knowing the weaning age on the hedonic evaluation.

## 2. Materials and Methods

### 2.1. Animals and Samples

A total of 26 *Arouquesa* animals were raised in the region of the breed and assigned to 4 groups, with combinations of weaning (W) at 9 or 5 months and slaughter (S) at 9 or 12 months: W9-S9, W9-S12, W5-S9, and W5-S12.

The animals were fed with dam milk until 3 months. From 3 months until the slaughter, the feeding included dam milk until the defined weaning age, ad libitum hay, ground corn, and a cereal-soya-based feed.

The animals were transported to the abattoir the day before slaughter, stunned with a captive bolt, and slaughtered, dressed, and chilled for 24 h in an accredited abattoir according to current EU regulations on the protection of animals at the time of killing [24]. The longissimus thoracis et lumborum muscle was collected, divided into two portions, one for sensory analysis and the other for physicochemical analysis, vacuum packed and aged until 7 days postmortem at 4 °C. A total of 26 samples were tested (7, W9-S9; 5, W9-S12; 6, W5-S9 and 8, and W5-S12).

### 2.2. Physicochemical Parameters

The pH was measured at 24 h (pH_24h_) postmortem with a penetration electrode accoupled to pH meter WTW 330i (Weilheim, Germany) after calibration with buffers of pH 4.01 and 7.00.

Meat colour parameters (L*, a*, b*) were measured on the meat surface with a Minolta Chroma Meter CR-310 (Osaka, Japan) and a D65 illuminant. The measurement was taken after 60 min of blooming by placing the samples in trays covered with polyethylene film at 4 °C.

The water holding capacity of meat was assessed by cooking losses; samples of approximately 90–100 g were placed individually in polyethylene bags in a water bath at 80 °C and cooked until an internal temperature of 71 °C. After being cooled for 15 min in an ice water bath, the samples were stored overnight at 4 °C. The samples were then dried with filter paper and weighed. The cooking losses were expressed as a percentage of the initial sample weight [25,26].

For the shear force determination, the meat samples used for cooking losses were cut into cuboid shape sub-samples (6 to 8 from each sample) of 1 cm^2^ cross-section and 3–4 cm in length. After room temperature equilibrium, samples were placed with fibres perpendicular to the direction of a Warner–Bratzler rectangular hole probe coupled to a TA.XT plus texturometer (Stable Micro Systems, Godalming, UK), with a load cell of 30 kgf, blade velocity set to 200 mm/min, and trigger force of 5 g. Maximum shear force values were recorded, and the values were expressed in N/cm^2^.

### 2.3. Sensory Analysis

Steaks (1.5 cm) were cut and cooked as previously described for cooking losses. Pieces of 2 × 2 cm were cut from the cooked steak. Two pieces were rolled up in aluminium foil and kept at 60 °C until served, which occurred less than 30 min from the end of cooking. Seventy consumers, recruited from the authors’ personal and professional contacts, performed a Check All That Apply (CATA) test. Less than 10% of the participants were students. The group was composed of 48% women, aged 19 to 66 years (38.3 ± 15.4). Education and occupation were diversified. Eighty-six percent were regular beef consumers, with 79% indicating consumption one to three times a week. Less than half (46%) were regular consumers of PDO beef. The tests were performed in a sensory analysis laboratory, with individual booths and uniform illumination. The samples were presented and identified with a three-digit number. The presentation of samples was randomised to avoid order effects. Spring water and salt were available to clean the mouth. Consumers were asked to evaluate the general appreciation of each sample on a 9-point hedonic scale and to mark in a list of 22 attributes those they considered to apply: aroma–cooked meat/beef broth; cooked fat (pleasant); flavour—cooked meat/beef broth; cooked fat (pleasant); sweet; bitter; sour; bloody; liver; hay; grass/vegetal; cardboard; fish; texture—tender; very tender; tough; very tough; fibrous; juicy; dry; disintegrate quickly in the mouth; takes time to chew. The vocabulary was adapted from the AMSA guidelines for beef sensory evaluation [26].

Viewing the evaluation of the knowledge on the weaning age for consumer appreciation, two samples were presented, identified with the weaning age of the animal. An introductory description was included in the test form “This meat comes from animals raised in a traditional system. They were suckling and fed on local grass, hay, and a cereal-based supplement. Some animals are weaned late, taking advantage of the mother’s entire natural lactation cycle (9 months), and others are weaned earlier (5 months)”. The meat samples were identified with “late weaning—9 months” and “early weaning—5 months”. The consumers were asked to evaluate the general appreciation of each sample on a 9-point hedonic scale, identical to the evaluation made in the first part of the testing session with anonymous samples.

### 2.4. Statistical Analysis

The physicochemical parameters and hedonic evaluation were compared by one-way ANOVA (*p* < 0.05). The Cochran’s Q test compared binary data from the CATA test. Attributes and samples were analysed by principal coordinate analysis (PCoA) and penalty analysis. The comparison between the hedonic evaluation of the meat presented anonymously and identified was compared by the Wilcoxon test. The statistics were calculated with XLStat, Addinsoft, and Paris.

## 3. Results and Discussion

### 3.1. Physicochemical Parameters

The results for physicochemical parameters are shown in Table 1.

No significant differences in pH_24h_ due to weaning slaughter age were observed.

For the colour parameters, the mean values of the four groups were 40.53, 24.97, and 7.90 for L*, a*, and b*, respectively, and did not differ significantly between groups. Bispo and colleagues [12] also reported the absence of differences associated with different weaning ages for L*, a*, and b*. The a* values observed in the present study are higher than those observed by the cited authors, 15.81 and 14.80, for 8- and 5.5-months weaning ages, respectively. The higher values we found might be due to differences in intramuscular fat content, myoglobin content, or meat ageing in the vacuum that promotes increasing a* and b* values [16]. During ageing, there is a reduction in mitochondrial oxygen consumption, resulting in more oxygen available for oxymyoglobin formation and enhanced meat blooming [27,28,29]. Comparing traditional weaning age (5 months) and early weaning (3 months), Blanco and colleagues [30] also did not find differences in L* and hue values in the meat of calves. Other authors [21] reported higher L* and b* values but the same a* in later weaning animals (7 vs. 3 months). In the present study, we did not find differences in any parameter due to the slaughter age. The meat of Holstein calves slaughtered at 12 months of age has the same L* but significantly higher values for a* and b* compared to calves slaughtered at 9 months [31].

Cooking losses did not differ significantly between groups regarding weaning or slaughter age. These results are consistent with previous studies in *Rubia Gallega* veal [12,16] that also did not find significant differences with weaning age. The cooking losses reported by these authors are higher (30%) than those found in the present work (17.30–21.88%). Pateiro and colleagues [21] also did not find differences in cooking losses related to weaning age. The values these authors reported for calves weaned before 3 months and at 7 months were 22.03 and 22.41%, respectively, which are similar to those found in the present work. Different methodologies, namely the temperature/time of cooking, may explain these differences. Considering the slaughter age, Cho and colleagues [31] have also not found differences in cooking losses in Holstein calves slaughtered at 9 and 12 months.

When animals were slaughtered at 9 months, the early weaning (5 months) resulted in a higher (*p* < 0.05) shear force than in those weaned at 9 months. The relationship between the weaning age and the shear force has contradictory results in previous studies. In the same sense of our results, Pateiro and colleagues [21] observed a higher shear force in veal with weaning at 3 months than when animals were weaned later (7 months). On the contrary, no influence of the weaning age on the shear force was observed in a Spanish breed veal meat [12].

The slaughter age did not influence the shear force or the same weaning age. Similar results were also observed by Florek and colleagues [22]. Other authors reported that meat from calves slaughtered at 12 months had a lower shear force than those slaughtered at 9 months [31]. It is usually accepted that older slaughter ages have a negative effect on meat tenderness, resulting from intramuscular connective tissue accumulation and the consequent mechanical strength increase, as well as the decrease in collagen heat solubility [32]. However, the 3 months of difference in the present study, from 9 to 12 months, are probably too short to allow the clear manifestation of these modifications, particularly in *Arouquesa* meat, which is considered very tender.

### 3.2. Sensory Analysis

**Figure 1:**
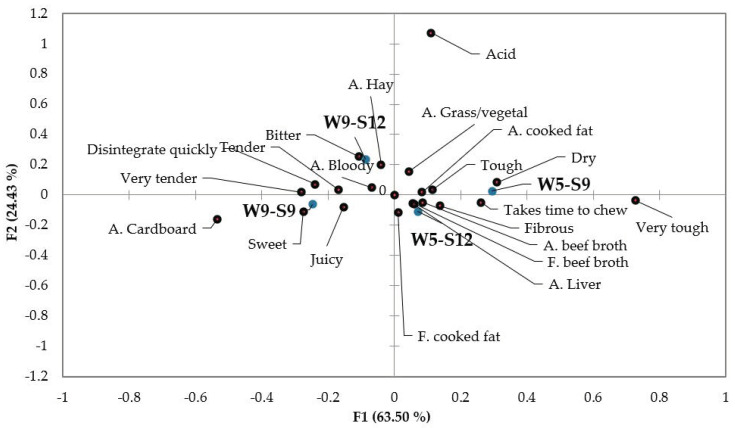
Attributes of *Arouquesa* meat tested (in blue) in the space defined by the first two factors (A.—Aroma; F.—Flavour).

The CATA results are presented in Table 2, and the relationship between attributes and the samples is illustrated in Figure 1. Out of the 22 attributes (Table 2), eight presented differences (*p* < 0.05) between the groups, namely the meat broth and fat aroma, sweet flavour, tender and very tender, very tough, juiciness, and ease of disintegration in the mouth. Meat from groups W9-S9 and W5-S12 had higher beef broth aroma and flavour notes. These groups have different weaning and slaughter ages, making it difficult to infer the specific effect. Animals slaughtered at 9 months and weaned at 5 and 9 months were distinct (*p* < 0.05) for tenderness/toughness, as well as juiciness and facility to chew (disintegrate quickly). That trend of differences found by the consumers performing the CATA test is coherent with the shear force results (Table 1). It is common to have a relationship between cooking losses and juiciness [33], but in the present study, the differences observed by consumers did not find a parallel in the gravimetric determination of cooking losses. Once the study was performed with consumers, with no specific training on texture evaluation, one can hypothesize that the pleasant textural proprieties were evaluated in the same sense [34], more tender and also juicier.

The PCoA results (Figure 1) show the relationship between the attributes evaluated by the CATA test and between the attributes and the meat groups. Attributes on the same plan side are related to each other in the same sense. Attributes on opposite sides of the plan are oppositely related. It is possible to observe that the texture parameters were discriminant, with tenderness, juiciness, and ease to disintegrate in the mouth on the left side of the plan, and tough, fibrous, dry and time to chew on the right side. Most of the aroma and flavour attributes are on the right side of the plan, together with the tough, dry and fibrous texture, indicating that the tenderer and juicier meat is less aromatic. The meat grouped with these sensory attributes were from animals with an earlier weaning age (W5-S9 and W5-S12). That relationship between toughness, dryness and fibrousness, and the aroma/flavour attributes was probably due to the feeding of the animals during the post-weaning period. The calves weaned at 5 months had been fed for 4 or 7 months with pasturage and a cereal-based supplement, depending on being slaughtered at 9 or 12 months. It is recognised that the meat flavour might be influenced by the feed composition, namely the type of fats it supplies and the abundance and type of terpenoids that can be stored in the meat and define its flavour [35,36].

The meat from the W9-S12 and W5-S12 groups was projected near the referential centre, indicating poor discrimination by the sensory attributes. These results suggest that the differences observed for weaning age when the animals are slaughtered at 9 months disappear when they are slaughtered at a later age.

Considering that the calves weaned at 5 months were fed with pasturage and supplemented with cereals, predominantly corn, and taking into consideration the importance of the protein quantity and quality at this growth phase [37], we can speculate that those animals weaned at 9 months had a more balanced protein intake. This availability of protein might result in an increased turnover of myofibrils. The growth-associated protein turnover enzymes are also associated with the meat’s postmortem tenderization. The high pre-slaughter proteolytic activity associated with increased protein turnover is thus associated with higher postmortem protein breakdown, with the expected consequences on meat tenderness [38].

**Figure 2:**
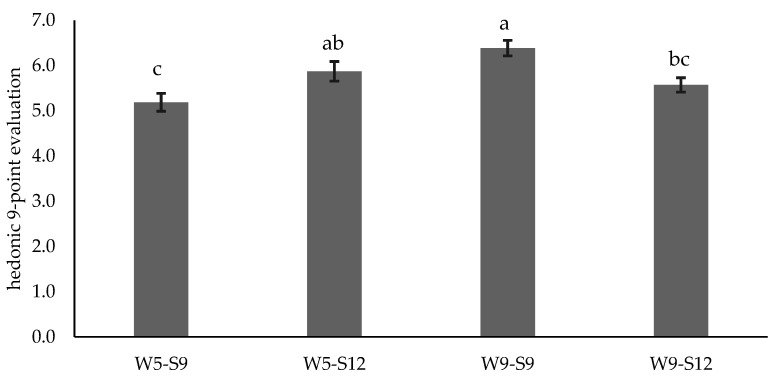
Hedonic evaluation of the four types of meats presented anonymously. Results are expressed as mean and standard error. Bars with different letters are different (*p* < 0.05).

The results of the hedonic evaluation of the meat from the four experimental conditions evaluated anonymously are presented in Figure 2.

The meat from the animals W9-S9 and W5-S12 was better appreciated, with mean classification on the 9-point scale of 6.39 and 5.87, respectively. These meats presented a higher proportion of references to beef broth aroma, flavour, and a tender and juicy texture. The influence of each sensory attribute on the hedonic evaluation was evaluated through penalty analysis. With this approach, the hedonic evaluations of samples presenting or not presenting an attribute are compared by subtraction.

**Figure 3:**
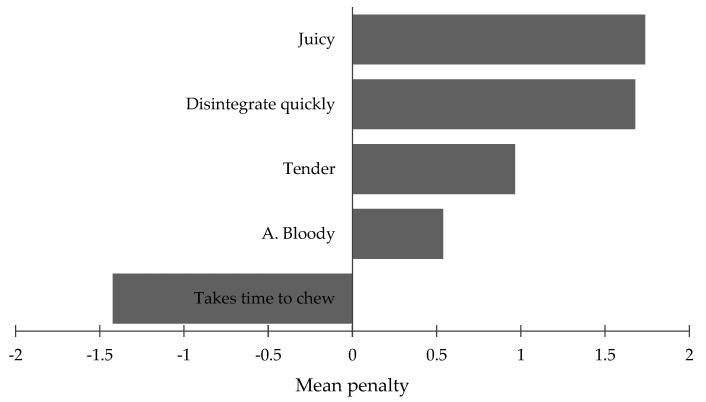
Mean penalties associated with specific attributes (A.—Aroma).

Some texture parameters were the main determinants of the higher hedonic evaluation (Figure 3). The figure only presents the attributes with a significant (*p* < 0.05) mean impact. On average, consumers indicating meat as juicy made a hedonic evaluation 1.7 points higher than those not indicating that characteristic. A similar appreciation trend was observed for the facility to disintegrate in the mouth, with tenderness and a bloody aroma. The time to chew reduced the hedonic evaluation by almost 1.5 points on the 9-point scale. Our results confirm a well-established trend between the texture attributes, particularly tenderness and juiciness, and beef acceptability [18,33,34].

The psychological factors influencing the consumer evaluation of meat are diverse [39]. In the present study, we tested the effect of identifying the weaning age, hypothesizing that the consumer would see longer suckling calves as a welfare advantage. The consumer’s psychological influence was tested in different contexts related to animal welfare, organic production, or fewer chemical additives. The information on the consumer’s psychological expectations results in a higher sensory appreciation [39,40,41,42,43]. When comparing the results from the hedonic evaluation of the same meat presented anonymously and identified with the weaning age (Table 3), we can observe that the evaluation was similar (*p* = 0.073) for the early weaning group but with a slight tendency to improvement in the evaluation of the identified samples. When comparing the results for the weaning at 9 months, the effect of information was evident, resulting in 57.1% of the consumers changing their hedonic evaluation for a higher score. These results indicate that the information on welfare-related issues improves meat acceptability, even in the general hedonic appreciation [40,41,42,43,44].

## 4. Conclusions

The weaning and slaughter age of the *Arouquesa* animals influence some meat characteristics. The early weaning at 5 months and slaughter at 9 months results in tougher meat, revealed by the higher shear force and the consumers’ evaluation in the CATA test. The meat from animals slaughtered at 9 months is more tender and juicier if they were suckling until the slaughter. When animals are slaughtered at 12 months, there is almost no difference between the weaning ages. Consumers’ hedonic evaluation of meat is influenced by the information on the weaning age, with an advantage for later weaning.

## Figures and Tables

**Table 1:** Physicochemical parameters of meat quality (mean and standard error).

Parameters	W5-S9	W5-S12	W9-S9	W9-S12	*p*
pH_24h_	5.64 (0.07)	5.56 (0.05)	5.63 (0.07)	5.60 (0.04)	0.798
L*	40.18 (2.02)	41.49 (0.64)	39.84 (1.48)	40.37 (2.03)	0.851
a*	23.95 (0.97)	26.29 (0.45)	23.96 (0.78)	25.52 (1.41)	0.140
b*	7.11 (0.84)	9.07 (0.36)	6.83 (0.79)	8.45 (1.05)	0.104
Cooking losses (%)	17.30 (1.82)	18.32 (0.34)	17.65 (0.85)	21.88 (2.93)	0.198
Shear force (N/cm^2^)	87.02 (3.89) b ^1^	64.13 (6.42) ab	62.47 (5.68) a	63.47 (8.15) ab	0.032

^1^ Means with different letters are different (*p* < 0.05).

**Table 2:** The proportion of consumers identifying each attribute.

Attributes	W5-S9	W5-S12	W9-S9	W9-S12	*p*
Beef broth aroma	0.53 a ^1^	0.73 b	0.56 ab	0.37 a	<0.0001
Cooked fat aroma	0.19	0.14	0.19	0.10	0.289
Beef broth flavour	0.43 ab	0.51 b	0.49 b	0.27 a	0.005
Cooked fat flavour	0.16	0.26	0.219	0.11	0.112
Sweet	0.07 a	0.20 ab	0.249 b	0.11 ab	0.009
Bitter	0.06	0.09	0.09	0.10	0.750
Acid	0.04	0.00	0.019	0.07	0.069
Tender	0.23 a	0.36 ab	0.46 b	0.29 ab	0.017
Very tender	0.10 a	0.14 ab	0.26 b	0.13 ab	0.044
Tough	0.14	0.24	0.13	0.14	0.244
Very tough	0.17 b	0.10 ab	0.03 a	0.03 a	0.005
Fibrous	0.19	0.29	0.17	0.13	0.112
Juicy	0.26 a	0.36 ab	0.50 b	0.21 a	0.001
Dry	0.24	0.26	0.11	0.16	0.087
Disintegrate quickly	0.19 a	0.33 ab	0.46 b	0.30 ab	0.006
Takes time to chew	0.30	0.29	0.20	0.13	0.055
Bloody	0.20	0.29	0.30	0.21	0.318
Liver aroma	0.10	0.14	0.11	0.07	0.514
Hay aroma	0.07	0.06	0.09	0.07	0.927
Grass/vegetal aroma	0.10	0.09	0.10	0.09	0.971
Cardboard aroma	0.01	0.04	0.10	0.03	0.081
Fish aroma	0.00	0.00	0.00	0.00	1.000

^1^ proportion followed by different letters are different (*p* < 0.05).

**Table 3:** Variation of the hedonic appreciation of anonymous and identified samples with age at weaning (5 or 9 months). Results are expressed in the percentage of responses in the indicated direction.

With Identification	W5	W9
Reduce	31.4	22.1
Improve	38.6	57.1
Maintain	30.0	20.8
Z (*p*)	−1.791 (0.073)	−3.492 (<0.001)

## Data Availability

The data presented in this study are available on request from the corresponding author.
